# Thrombolysis use in STEMI patients in Central Asia: lessons from the Bukhara region of Uzbekistan

**DOI:** 10.3389/frhs.2026.1797882

**Published:** 2026-04-24

**Authors:** Dildora Aslonova, Rakhmon Amanov, Vokhid Zokirov, Gulnora Fayzillayeva, Muzayyam Nekova, Maxtob Farmanova, Munisa Gulova, Fotima Khodjaeva

**Affiliations:** 1 Department of Therapeutic Sciences, Zarmed University, Bukhara, Uzbekistan; 2 Republican Specialised Scientific-practical Medical Center of Cardiology Bukhara Territorial Branch, Bukhara, Uzbekistan; 3 Department of Preclinical Sciences, Zarmed University, Bukhara, Uzbekistan; 4 Department of Infectious Diseases and Infectious Diseases of children, Bukhara State Medical Institute, Bukhara, Uzbekistan; 5 Department of Socio-Political Sciences, Bukhara State University, Bukhara, Uzbekistan

**Keywords:** acute coronary syndrome, Central Asia, healthcare disparities, STEMI, thrombolysis

## 1 Introduction

### 1.1 Acute coronary syndrome: a global problem and regional challenges

Acute coronary syndrome (ACS)—including ST-segment elevation myocardial infarction (STEMI) and non-ST-segment elevation acute coronary syndrome (NSTEMI)—remains one of the leading causes of death worldwide. According to the World Health Organization, cardiovascular diseases claim the lives of more than 17.9 million people each year, accounting for 32% of global deaths (1). Acute myocardial infarction occupies a special place in this, and if not treated promptly and effectively, it can have a mortality rate of up to 30% (2).

Although primary percutaneous coronary intervention (PCI) is the gold standard for the treatment of STEMI in developed countries (3), fibrinolytic therapy remains the main reperfusion strategy in low- and middle-income countries due to the limited availability of PCI centers (4). However, global studies show that even fibrinolytic therapy is not sufficiently used in resource-limited settings (5).

Uzbekistan is characterized by a high prevalence of cardiovascular diseases. The country has a population of more than 35 million, most of whom live in rural areas, which makes access to specialized medical care difficult (6, 7). In addition, the pre-hospital medical care system, including emergency medical services, is still in its infancy.

In this article, we aimed to analyze the current state of treatment of patients with ACS in the Bukhara region of Uzbekistan, identify existing health system and policy barriers, and propose evidence-based policy recommendations. A comparison of key ACS management indicators between global targets, developed countries, India, and the Bukhara region is presented in Table 1. We argue that improving ACS outcomes requires not only clinical interventions but fundamental changes in health policy, governance structures, and resource allocation strategies.

**Table 1:** Comparison of ACS management indicators.

Indicator	Global Target (ESC/AHA)	Developed Countries	India (CREATE)	Bukhara Region (2025)
Reperfusion rate in STEMI	>90%	85-95%	58.5%	**31.3%**
Primary PCI rate	Preferred	70–85%	8.6%	Limited
Door-to-needle time	<30 min	25–40 min	50 min	N/A
In-hospital mortality	<5%	4–7%	8.6%	6.3%
Fibrinolytic agent	Tenecteplase	Tenecteplase	Streptokinase/ Tenecteplase	Streptokinase only
STEMI network	Recommended	Established	TN-STEMI model	Not established

ESC, European Society of Cardiology; AHA, American Heart Association; PCI, percutaneous coronary intervention; STEMI, ST-elevation myocardial infarction; N/A, not available. Sources (10, 14, 15, 22, 23).

Bold values indicate data from the Bukhara Region (2025), representing the primary findings of the present study.

## 2 Current status of acute coronary syndrome management in Uzbekistan

### 2.1 Challenges in healthcare infrastructure

Central Asian countries, including Uzbekistan, face significant challenges in adapting their Soviet-era healthcare systems to modern requirements (8). Cardiovascular disease is the leading cause of death in the five Central Asian republics, with more than 1.5 million cases of acute and chronic cardiovascular disease reported annually in Uzbekistan (9). The cardiovascular system is largely organized around regional and district hospitals, with primary percutaneous coronary intervention (PCI) capabilities limited to specialized centers in large cities, and access to thrombolytic therapy remains limited in most institutions (9).

There are 12 regions in Uzbekistan, most of which have very limited catheterization laboratories providing PCI services. According to the European Society of Cardiology (ESC) recommendations, primary PCI in STEMI patients should be performed within 120 min (10). However, due to geographical distances, inadequate transport infrastructure and limited ambulance capacity, achieving this time frame is almost impossible in rural areas (11).

In addition, the ability to perform electrocardiography (ECG) and diagnose STEMI in the pre-hospital setting is limited. Many ambulance crews do not have a 12-lead ECG machine, which slows down early diagnosis and referral to the appropriate hospital (12).

### 2.2 Current situation in thrombolysis usage

Fibrinolytic therapy remains the main reperfusion strategy for STEMI patients in settings where PCI is not possible (13). According to international recommendations, if primary PCI cannot be performed within 120 min, fibrinolytic therapy should be initiated within the first 12 h, preferably within the first 30 min (10).

However, the data indicate that fibrinolysis is not being used sufficiently. According to the data of the Bukhara Regional Health Department for November 2025, a total of 1,070 ACS and 912 AMI patients were registered in the region (14). Of these, 278 patients had ACS with ST-elevation (STEMI), but only 86 patients (31.3%) received thrombolytic therapy. This is significantly lower than the 92.7% reperfusion rate (84.8–97.5%) reported across 29 countries in the 2021 European Society of Cardiology STEMI Registry (15). It is noteworthy that streptokinase is the only fibrinolytic agent used in all medical institutions in the region (14). However, current recommendations favor fibrin-specific agents such as tenecteplase or alteplase, as they are more effective and have fewer side effects (16). The use of streptokinase, although economically feasible, poses problems with the risk of allergic reactions and restrictions on repeated use (17).

### 2.3 Differences between urban and rural areas

The analysis of the data shows that there are significant differences between urban and rural areas. In the branches of the major medical institutions in Bukhara, the Republican Specialized Scientific and Practical Medical Center of Cardiology (RISCMC) and the Republican Scientific Center of Emergency Medical Care (RSCEM), the use of thrombolysis was almost non-existent (0%), although these centers admitted the largest number of STEMI patients (14). This situation can be explained by the availability of PCI in these centers or the strategy of timely referral of patients to PCI centers.

Interestingly, the use of thrombolysis in rural areas is relatively high, averaging 34.2% (14). The highest rates were recorded in Jondor district (73.8%), Vobkent district (95.2%), which may indicate that some NSTEMI patients were also treated with thrombolysis. This difference is explained by the lack of access to PCI centers in rural areas and the fact that fibrinolytic therapy is the only reperfusion method. The overall mortality rate in the region was 6.3%, which is close to the 4%–5% in-hospital mortality rate reported in modern registries in developed countries (18), although some districts (Bukhara district—10.5%, Gijduvan—10.2%, Peshku—9.8%) have significantly higher mortality rates (14). These differences may partly reflect inequalities in access to care. However, patient-level factors such as age, infarction location, comorbidities, and time to treatment may also contribute to the variation in observed mortality rates. The lack of patient-level data in our analysis limits our ability to determine the relative contribution of each factor.

### 2.4 Anomaly in the karakul district: An example of protocol violation

The reported thrombolysis rate in the Karakul District of 110%, meaning more patients received thrombolysis than were diagnosed with STEMI, requires thorough investigation, not just a cursory mention. This anomaly reveals fundamental problems in diagnosis, documentation, and protocol adherence that likely exist throughout the regional healthcare system.

We investigated this finding through discussions with colleagues in the district and a review of available documentation. Several non-mutually exclusive explanations could explain this alarming statistic.

First, misclassification of diagnoses is likely occurring. In our assessment, the most likely explanation is that some patients with NSTEMI or even unstable angina were misdiagnosed as STEMI and subsequently received thrombolysis. Physicians in community hospitals often lack access to serial troponin testing, and their ECG interpretation skills vary significantly. We have observed cases where nonspecific ST-T segment changes, left bundle branch block, or even early repolarization patterns were misinterpreted as ST-segment elevation myocardial infarction. Without cardiologist oversight and under pressure to “do something” for patients with chest pain, some physicians may prescribe thrombolysis based on clinical suspicion rather than clear ECG criteria.

Second, documentation and reporting errors may contribute. The regional medical statistics system relies on manual reporting by district hospitals. We suspect that some institutions may underreport STEMI diagnoses while accurately reporting thrombolysis, creating an artificial discrepancy between the numerator and denominator. Alternatively, the diagnosis of patients initially classified as NSTEMI who subsequently developed STEMI (and who received thrombolysis) may not be updated in the reporting system. The lack of integrated electronic medical records makes accurate tracking extremely difficult.

Third, protocol violations may be intentional. We must consider the troubling possibility that some physicians knowingly administer thrombolysis to patients with NSTEMI. This may be due to a misunderstanding of guidelines, the belief that “some treatment is better than no treatment”, or pressure from patients or their families to pursue aggressive intervention. In resource-limited settings where PCI is unavailable, physicians may consider fibrinolysis the only available “active” treatment, even when guidelines recommend only medical therapy.

This finding is not simply a statistical curiosity—it represents a patient safety concern. Thrombolysis in patients with NSTEMI is not recommended by any major guidelines because it does not improve outcomes and increases the risk of bleeding (19, 20). The TACTICS-TIMI 18 trial and other landmark studies have shown that patients with NSTEMI benefit from early invasive strategies or medical therapy, not fibrinolysis.

Providing thrombolysis to patients with STEMI exposes them to significant harm without the expected benefit:

− 0.5–1% risk of intracranial hemorrhage—potentially fatal or disabling
− 4–6% risk of major bleeding—requiring blood transfusion or intervention
− No reduction in mortality—unlike STEMI, where timely thrombolysis saves lives
− Potential delay in appropriate treatment for bleeding complications

If even 10–20 patients with STEMI in the Karakul district receive inadequate thrombolysis, we can expect 1–2 cases of major bleeding and possibly one stroke or death directly related to this practice. These iatrogenic consequences are preventable.

### 2.5 What does this say about the system as a whole?

In our opinion, the Karakul anomaly is likely not unique—it is simply the most illustrative example of systemic problems existing throughout the region. Other districts with thrombolysis rates of 70–95% may also treat some STEMI patients, but not enough to exceed 100%. We hypothesize that inappropriate thrombolysis occurs throughout the system with varying frequency.

This finding highlights several critical systemic failures:

− Lack of diagnostic standards: There are no mandatory criteria for diagnosing STEMI before administering thrombolysis.
− Lack of monitoring: No cardiologist examination or telemedicine consultation is available.
− Lack of audit mechanisms: Inappropriate treatment goes undetected and unreported.
− Lack of training: Insufficient ECG interpretation skills.
− Lack of feedback: Physicians never know whether their diagnoses were correct.

### 2.6 Urgent investigation and corrective action

We urge the regional health department to conduct an urgent clinical audit of all thrombolysis cases in Karakul and other high-incidence districts. This audit should determine:

− How many patients with STEMI or without ACS received thrombolysis?
− What were the clinical outcomes, including bleeding complications?
− What ECG findings informed the treatment decision?
− Was a pretreatment consultation by a cardiologist provided?

Based on the audit findings, immediate corrective actions should include:

− Required ECG transmission to a regional cardiologist before any thrombolysis
− A standardized STEMI diagnostic checklist that must be completed and documented
− Real-time analysis of all thrombolysis cases within 24 h
− Feedback to physicians on diagnostic accuracy and outcomes
− Refresher training programs for institutions with high rates of inappropriate treatment

We emphasize that our goal is not to blame individual physicians who may be doing their best under difficult circumstances. Rather, we seek to highlight a systemic failure that puts both patients and physicians at risk. The Karakul anomaly should serve as an alarm signal for regional and national health authorities.

There are a number of successful experiences in improving the care of patients with ACS in resource-limited countries. These experiences provide valuable lessons for Uzbekistan.

### 2.7 The Indian experience: Tamil Nadu STEMI network

As a country of over 1.4 billion people, mostly living in rural areas, India faced similar challenges to Uzbekistan (21). According to the CREATE registry, only 58.5% of STEMI patients in India received reperfusion therapy, with only 8.6% undergoing primary PCI (5).

The STEMI network (TN-STEMI) established in 2010 in the state of Tamil Nadu has fundamentally changed this situation (22). The main principles of this program were as follows:

First, a “hub-and-spoke” model was introduced—a network of several peripheral hospitals (spokes) was established around a central PCI center (hub). Patients diagnosed with STEMI in peripheral hospitals were immediately started on fibrinolytic therapy and then transferred to a PCI center (pharmaco-invasive strategy) (23).
Second, a mobile ECG transmission system was introduced. ECG images obtained by ambulance crews were transmitted to the central hospital via smartphone and analyzed remotely by a cardiologist. This significantly reduced the time from “first medical contact to diagnosis” (24).
Third, the drug tenecteplase was introduced. Compared to streptokinase, this drug is characterized by a single bolus injection, high fibrin specificity, and fewer hemorrhagic complications (25).

As a result, in-hospital mortality decreased from 8.2% to 4.5%, and the percentage of patients receiving reperfusion increased from 60% to 92% in the program areas (23).

### 2.8 The Brazilian experience: telemedicine networks

Brazil is also a country that faces the challenges of a large geographical area and uneven health infrastructure. The Telemedicine Network (TNMG) implemented in the state of Minas Gerais covers more than 1,000 municipalities (26). In this system, ECGs performed in rural health centers are transmitted via the Internet to a central station, where they are analyzed and interpreted by a cardiologist within 10 min. The results of the 10-year program have shown that the “symptom-to-treatment” time for STEMI patients has been reduced by an average of 40% (27).

In addition, in Brazil, the national program “Linha do Cuidado do Infarto” (Infarction Care Line) has standardized STEMI protocols across all states and centralized the provision of fibrinolytic drugs (28).

### 2.9 The South African experience: efficiency in resource-constrained settings

The South African health system is characterized by a large disparity between the secondary—private—and public sectors (29). According to the ACCESS-SA registry, 65% of STEMI patients in public hospitals received fibrinolytic therapy, with an average door-to-needle time of 60 min (30).

Interestingly, in the resource-constrained public sector, a strategy of “task-shifting” has been used effectively—specially trained nurses are authorized to initiate fibrinolytic therapy (31). This approach has helped to reduce reperfusion time in conditions of physician shortages.

### 2.10 Experience from Russia and the CIS

In Russia, the Vascular Centers program has been implemented since 2008 (32). Within the framework of this program, at least one vascular center has been established in each region, where PCI services are provided 24/7. In addition, telemedicine links have been established with district hospitals.

In Kazakhstan, the STEMI Kazakhstan program has been launched since 2016 (33). Initial results show that the reperfusion rate in large cities has reached 75%, but in rural areas this figure still remains around 40%.

### 2.11 Pharmaco-invasive strategy: the optimal approach for resource-limited areas

The general conclusion of all the above experiences is that in areas with limited PCI capabilities, the pharmaco-invasive strategy is the most effective approach (34). This strategy consists of the following steps:

1. Rapid diagnosis of STEMI in the primary care setting (based on ECG)
2. Immediate initiation of fibrinolytic therapy if there are no contraindications
3. Transfer to a PCI center within 3–24 h of fibrinolysis and coronary angiography
4. If necessary, perform PCI

The results of the STREAM study showed that a properly implemented pharmaco-invasive strategy provides similar clinical outcomes compared to primary PCI (35). This is especially relevant in the conditions of Uzbekistan, since it is difficult to establish PCI laboratories in all district centers in a short time.

### 2.12 Why is thrombolysis underused?

As physicians working in the Bukhara healthcare system, we have firsthand experience with the barriers hindering the adequate use of thrombolysis. Based on our experience, we have identified five interrelated factors that explain the current situation.

In our view, the most significant barrier is physicians' reluctance to prescribe fibrinolytic therapy due to fear of hemorrhagic complications. Many physicians in district hospitals have witnessed or heard of cases of fatal intracranial hemorrhage following thrombolysis, creating a culture of excessive caution. Unlike Western countries, where clear protocols protect physicians who follow recommendations, Uzbekistan lacks a clear medico-legal framework that protects physicians from liability when evidence-based treatment leads to complications. Consequently, many physicians prefer to “do nothing and transfer the patient to another hospital” rather than “treat and risk being held accountable”. This approach to defensive medicine directly contributes to the low thrombolysis rates we see.

Although streptokinase is theoretically available in district hospitals, in practice, it's much more complicated. We frequently see shortages lasting weeks or even months, especially in remote areas. The centralized procurement system creates delays: when a hospital runs out of supplies, the process of requesting, approving, and delivering new batches can take 4–6 weeks. Furthermore, proper cold chain conditions are essential for storing streptokinase, and unstable power supplies in some rural facilities compromise the integrity of the drug. Hospital administrators, unsure of the efficacy of stored streptokinase, may be hesitant to use it. In our experience, 30%–40% of district hospitals may have insufficient or questionable supplies of fibrinolytic agents at any given time.

In Uzbekistan, there is no national protocol for the treatment of ST-segment elevation myocardial infarction (STEMI). Each medical institution develops its own approach, resulting in significant variations in practice. We have observed that many physicians in district hospitals, especially older physicians trained during the Soviet era, are unsure of their ability to administer thrombolysis. They were trained during an era when fibrinolytic therapy was rarely available, and subsequent advanced training has proven insufficient to change established practices.

Furthermore, the decision to administer thrombolysis often requires consultation with a cardiologist, who may not be available in district hospitals, especially during night shifts and weekends. This consultation requirement, while intended to ensure appropriate patient selection, creates delays that place patients outside the therapeutic time window. We have observed cases where patients were admitted within 2 h of symptom onset but received thrombolysis only 6–8 h later due to consultation delays, or did not receive it at all.

The current healthcare financing system inadvertently hinders the provision of thrombolysis at the district level. District hospitals receive fixed budgets regardless of the intensity of treatment, which creates no financial incentive for complex interventions. Meanwhile, regional tertiary care centers are reimbursed based on patient volume and procedure complexity, which encourages them to accept patients from other facilities rather than maintain treatment at the district level.

We are witnessing a deeply ingrained “referral culture,” where district physicians view their role as one of stabilization and patient transfer, rather than definitive treatment. The system's implicit message is: “Send the patient to the regional center; the specialists are there”. This culture persists even when long transportation distances make timely PCI impossible and when fibrinolysis at the district level would clearly benefit the patient.

Finally, we must acknowledge that many patients present too late for effective reperfusion therapy. In our experience, the average time from symptom onset to hospital arrival in rural Bukhara exceeds 6 h—significantly longer than the optimal time window for fibrinolysis. Contributing factors include low public awareness of myocardial infarction symptoms, a cultural tendency to wait and see if symptoms resolve, a lack of private transportation in rural areas, and a distrust of district hospitals, leading some patients to bypass them entirely, preferring to travel to regional centers.

However, we emphasize that patient delays do not fully explain the low rates of thrombolysis. Even among patients who arrive within the guideline-recommended time window, many do not receive fibrinolytic therapy. The systemic barriers we described are the primary reasons for underutilization.

In our frank assessment, the current system is designed in such a way that low thrombolysis rates are virtually inevitable. The combination of fearful physicians, unreliable drug supplies, lack of protocols, misaligned incentives, and a culture of patient referrals creates a self-perpetuating cycle of undertreatment. Individual physicians cannot address this problem through improved knowledge or motivation alone; structural changes are required.

We believe that without addressing these root causes, any intervention—whether training programs, drug donations, or information campaigns—will have limited and unsustainable impact.

## 3 Discussion

Data from the Bukhara region illustrate not just a clinical problem, but a fundamental issue within the healthcare system. The 31.3% thrombolysis rate reflects not gaps in physician knowledge, but rather systemic barriers: procurement policies that exclude modern fibrinolytics, a lack of prehospital care protocols, fragmented patient referral systems, and a lack of accountability mechanisms for quality of care.

Addressing these issues requires coordinated action at multiple levels of government. The Ministry of Health must develop a policy framework and amend regulations; regional health departments must implement network structures and allocate resources; and healthcare facility administrators must implement protocols and monitor quality. Without such multi-level collaboration, isolated measures—such as training programs or equipment procurement—will fail to achieve sustainable improvement.

International experience shows that resource constraints need not impede progress. In India, a program in Tamil Nadu achieved significant mortality reductions with modest investment by leveraging existing infrastructure, implementing task-shifting policies, and establishing clear accountability structures. Uzbekistan could adapt these approaches to its own context, potentially achieving similar results within 3–5 years if appropriate policies are implemented. However, several barriers could hinder reforms. Bureaucratic inertia, competing health priorities, and limited health system management capacity pose challenges to their implementation. Successful reform will require sustained political commitment, potentially catalyzed by support from professional cardiology associations, patient groups, and international health partners. Pilot programs in receptive regions, such as Bukhara, could collect local data to support national scaling up of reforms.

## 4 Policy recommendations and implementation framework

Based on international data and analysis of the local context, we propose the following policy recommendations, organized by responsible stakeholders, required actions, and estimated resource requirements.

### 4.1 National policy changes (ministry of health)

First, the Ministry of Health should revise the National Essential Medicines List to include tenecteplase alongside streptokinase. Although tenecteplase costs approximately $500-$800 per dose compared to $50-$100 for streptokinase, its single bolus administration reduces healthcare staff time. It allows for its use in the prehospital setting, potentially offsetting the costs by reducing complications and shortening hospital stays. A phased implementation, initially targeting large centers, would cost approximately $2-$3 million per year initially.

Second, the Ministry should establish a National STEMI Registry through mandatory reporting requirements. This registry will enable quality monitoring, identify low-performing facilities, and provide data for evidence-based policy adjustments. Implementation costs are estimated at US$200,000–300,000 for software development and US$50,000 per year for maintenance, with potential funding from international health partners such as the WHO or the World Bank's Health Systems Strengthening Program.

Third, regulatory amendments should allow paramedics and trained nurses to administer fibrinolytic therapy under physician supervision via telemedicine. This task-shifting policy requires updating the Law on the Protection of Citizens' Health and developing standardized training protocols through the Ministry's Department of Medical Education.

### 4.2 Implementation at the regional level (regional health departments)

Regional health departments should establish formal STEMI referral networks using a center-periphery model. Each region should designate one facility capable of performing PCI, with 24-hour catheterization laboratory availability, linked to district (peripheral) hospitals through standardized referral protocols. The Bukhara Regional Health Department could pilot this model, with the Regional Cardiology Center serving as the hub.

Investment in telemedicine infrastructure is essential. Each district hospital requires ECG transmission capability (approximately $5,000 per facility for equipment and communications) and a regional interpretation center staffed by cardiologists. For the 13 districts of the Bukhara region, total infrastructure costs are estimated at $100,000–$150,000, which could potentially be financed through the regional health budget or international development assistance.

### 4.3 Facility-level protocols (hospital administrators)

Hospital administrators should implement standardized protocols for patient admission-to-treatment time with a target time of less than 30 min. This requires pre-stocking fibrinolytic drugs in emergency departments, having standardized orders to allow immediate treatment initiation, and eliminating administrative barriers such as the need for specialist consultation before thrombolysis. Quality improvement groups should conduct monthly reperfusion time audits and report the results to regional health departments.

### 4.4 Financing mechanisms and sustainability

Sustainable financing requires multiple funding sources. The government's guaranteed benefit package should explicitly cover the cost of fibrinolytic therapy, ensuring that patients do not face additional out-of-pocket costs. Centralized procurement through the Pharmaceutical Agency of Uzbekistan could reduce the cost of tenecteplase by 30%–40% through bulk purchasing agreements. International partnerships with organizations such as the Stent for Life Initiative, which has supported the development of ST-segment elevation myocardial infarction treatment networks in over 20 countries, could provide technical assistance and initial capital investment.

A cost-effectiveness analysis under similar conditions shows that increasing reperfusion rates from 31% to 80% could prevent approximately 50–70 deaths annually in the Bukhara region alone, with the estimated cost per life-year saved being $1,500–2,500—well below the cost-effectiveness threshold based on Uzbekistan's GDP. These economic arguments should be presented to the Ministry of Finance to justify budget allocations.

## 5 Study limitations and scope

We acknowledge several important limitations of the data presented in this article, which readers should consider when interpreting our findings and recommendations.

The regional data we present only cover November 2025—a single month that may not reflect annual patterns. Several factors may cause monthly variations in ACS treatment:

### 5.1 Seasonal effects

Cardiovascular events exhibit well-documented seasonal variations, with higher rates in the winter months (36). November marks the beginning of the cold season in Uzbekistan, potentially reflecting a transition period rather than peak or baseline rates. Thrombolysis use may differ during the summer months, when patient volume is lower and staff availability may fluctuate due to holiday schedules.

### 5.2 Drug supply variations

As discussed previously, the supply of fibrinolytic agents is variable. November data may reflect a period of adequate streptokinase availability or, alternatively, a period of shortage. Depending on supply timing, a facility that did not perform thrombolysis in November may have treated several patients in October or December.

### 5.3 Reporting cycle impact

Year-end reporting pressure may impact data quality. Some facilities may under- or over-report to meet estimated targets. We are unable to determine whether November 2025 represents typical reporting accuracy or an anomaly.

### 5.4 Staffing variations

The presence or absence of a single motivated physician at a district hospital can significantly impact thrombolysis rates. Staffing changes, hospital assignments, or temporary assignments can cause significant monthly fluctuations that our single-month data cannot capture.

To truly understand patterns in ACS treatment, longitudinal data spanning at least 12 months, and ideally several years, is necessary. We recommend that future analyses examine annual trends to identify consistent patterns vs. random fluctuations.

Bukhara is one of 14 regions in Uzbekistan, and our findings may not generalize to other regions. Several factors make Bukhara potentially unrepresentative:

### 5.5 Geographical characteristics

Bukhara is a relatively flat, desert region in western Uzbekistan. Mountainous regions (such as Surkhandarya or Namangan) face different transportation challenges that may further delay patient access to care. In contrast, the city of Tashkent has several centers capable of performing PCI (percutaneous coronary intervention), and they likely have very different treatment regimens.

### 5.6 Healthcare infrastructure

Bukhara has a regional cardiology center with PCI capability. Regions without centers capable of performing PCI may experience higher rates of thrombolysis (since it becomes the only option) or lower rates (if physicians simply refer all patients without attempting reperfusion). We cannot assume that the 31.3% rate observed in Bukhara applies elsewhere.

### 5.7 Economic factors

Regional economic development varies significantly across Uzbekistan. Wealthier regions may have better-equipped hospitals, more reliable drug supplies, and higher physician retention rates. Bukhara's status as a middle-income country may not reflect the situation in poorer or wealthier regions.

### 5.8 Historical and cultural factors

Patterns of medical practice are influenced by local traditions, educational institutions, and professional networks. The medical culture in Bukhara, shaped by its specific educational institutions and professional leaders, may differ from that of other regions.

We conducted our analysis in the context of Central Asia because the healthcare system challenges we described—Soviet-era infrastructure, limited access to PCI, reliance on fibrinolysis, and urban-rural disparities, which are common in Kazakhstan, Kyrgyzstan, Tajikistan, and Turkmenistan. Published data suggest similar challenges in ACS treatment across the region (8, 9, 33). Our goal was to present Bukhara as a case study illustrating regional patterns, rather than claiming that our data directly reflect the situation in all Central Asian countries.

We cannot confirm that thrombolysis rates in Bukhara reflect rates in Almaty, Bishkek, Dushanbe, or Ashgabat. Each country has its own healthcare financing systems, drug regulations, and medical education traditions. Readers should interpret our specific statistics (31.3% thrombolysis rate, 6.3% mortality) as applicable only to the Bukhara region. Our policy recommendations, while potentially relevant for all of Central Asia, will require adaptation to the specific context of each country.

## 6 Limitations in data quality

We must also acknowledge potential limitations in the underlying data quality:

Passive surveillance system: Data are obtained from routine administrative reporting rather than a prospective clinical registry. Diagnostic accuracy, case identification, and outcome tracking may be incomplete. Some STEMI cases may remain unreported, particularly patients who died before reaching the hospital or were treated in private clinics outside the national reporting system.

### 6.1 Lack of patient-level data

We present summary statistics without access to individual patient records. We are unable to analyze which patient subgroups received thrombolysis, the time intervals to treatment initiation, the frequency of contraindications, or individual outcomes. This limits our ability to determine whether low thrombolysis rates reflect appropriate patient selection (many contraindications) or a system failure (eligible patients not receiving treatment).

### 6.2 Outcome measurement

Mortality rates represent only in-hospital deaths. We lack data on 30-day mortality, a standard outcome measure in ACS registries. Patients discharged alive but who die shortly thereafter will not be counted, potentially leading to an underestimation of true mortality.

## 7 Implications of the findings

Given these limitations, we have sought to formulate our findings appropriately:

We present the Bukhara data as a case study illustrating broader issues, not as definitive regional statistics.
Our policy recommendations are formulated as proposals requiring local adaptation, not as universal prescriptions.
We call for the creation of adequate registries precisely because current data quality is insufficient for reliable analysis.
We acknowledge that further research in different regions and time periods is needed to confirm our observations.

Despite these limitations, we believe the data we present are valuable. Even imperfect data showing a 31.3% reperfusion rate—with recommendations for >85%—reveal a significant treatment gap that deserves attention. Qualitative findings based on our clinical experience complement quantitative data and are not dependent on statistical precision. Our goal is to stimulate discussion and policy action, not to provide definitive epidemiological estimates.

We encourage researchers in other regions of Central Asia to conduct similar analyses to obtain a more complete picture of ACS treatment in the region.

## 8 Conclusion

Data from the Bukhara region reveal a critical treatment gap: only 31.3% of patients with STEMI receive reperfusion therapy, significantly below the international target of 85%. These preventable deaths are due to health system failures, not to the shortcomings of individual physicians. Based on our analysis, we urge specific stakeholders to take the following actions:

The Ministry of Health should revise the National Essential Medicines List to include tenecteplase, develop a mandatory National STEMI Treatment Protocol, and establish a National ACS Registry for quality monitoring. Regulatory amendments should allow trained nurses and paramedics to initiate fibrinolytic therapy under telemedicine supervision.

Regional health departments should immediately conduct clinical reviews of thrombolysis cases in disadvantaged areas (especially in Karakul), establish center-to-periphery STEMI care networks, and ensure an uninterrupted supply of fibrinolytic agents with proper cold chain storage.

Based on our analysis, we urge specific stakeholders to take the following actions:

The Ministry of Health should revise the National Essential Medicines List to include tenecteplase, develop a mandatory National STEMI Treatment Protocol, and establish adequate cold chain storage systems.

Hospital leaders should set targets for admission-to-treatment times of less than 30 min, ensure pre-positioning of fibrinolytic agents in emergency departments according to standard orders, and eliminate administrative barriers that delay treatment initiation.

International organizations (WHO, World Bank, Stent for Life Initiative) should provide technical assistance in developing a STEMI treatment network, support pilot programs, and facilitate access to affordable tenecteplase through bulk procurement.

Medical schools and professional associations should strengthen training in ECG interpretation, develop national STEMI treatment guidelines adapted to local resources, and advocate for policy reform.

Experiences in India, Brazil, and South Africa demonstrate that significant improvements are achievable within 3–5 years with dedicated leadership and modest investment. The estimated annual cost of $2–3 million to develop a national STEMI treatment network is modest compared to the 50–70 lives that could be saved in each region annually.

We presented evidence, identified barriers, and proposed concrete solutions. Every month of delay costs lives that could be saved. Patients in Uzbekistan deserve the same chance of survival as patients anywhere else in the world.

We believe that the analysis and proposals presented in this article will spark a constructive discussion in the field of cardiovascular medicine in Uzbekistan and ultimately lead to positive changes in the lives of patients.
